# Prognostic value of the post-operative red blood cell distribution width in patients with rectal cancer with neoadjuvant chemoradiation followed by surgery

**DOI:** 10.1042/BSR20201822

**Published:** 2020-12-21

**Authors:** Yingkun Ren, Zhiling Wang, Jianguo Xie, Peijun Wang

**Affiliations:** 1 General Surgery, Affiliated Cancer Hospital of Zhengzhou University, Zhengzhou, Henan province, China; 2 Department of SICU, Affiliated Children's Hospital of Zhengzhou University, Henan Children’s Hospital, Zhengzhou, Henan province, China

**Keywords:** neoadjuvant chemotherapy, prognosis, rectal cancer, red-cell distribution width

## Abstract

**Purposes**: Several studies have reported that elevated red cell distribution width (RDW) is related to poor prognosis in several cancers; however, the prognostic significance of perioperative RDW in patients with rectal cancer that received neoadjuvant chemoradiation therapy (NACRT) is unclear.

**Methods:** A total of 120 patients with rectal cancer who received NACRT followed surgery were retrospectively reviewed from Affiliated Cancer Hospital of Zhengzhou University between 2013 and 2015. Data for peripheral blood tests prior to the initiation of NACRT, before surgery and first chemotherapy after surgery were collected, respectively. The optimal cutoff values of RDW were determined by ROC analysis, respectively. The relationship between RDW and the prognosis of patients was evaluated by the Kaplan Meier method, respectively.

**Results**: The post-operative RDW^High^ patients had significantly worse 5-year overall survival (OS, *P*=0.001) and disease-free survival (DFS, *P*<0.001) than the post-operative RDW^Low^ patients, respectively. Whereas high pre-operative RDW was the only marker correlated with worse DFS (*P*=0.005) than the pre-operative RDW^Low^ patients, no relationship was found between pre-RDW and prognosis (OS, *P*=0.069; DFS, *P*=0.133). Multivariate analysis showed post-operative RDW had better predictive value than pre-RDW and pre-operative RDW.

**Conclusion**: Post-operative RDW might be a useful prognostic indicator in patients with rectal cancer received neoadjuvant chemoradiation.

## Introduction

Colorectal cancer (CRC) is the third leading cause of cancer-related deaths in the world [1], rectal cancer accounts for 30.7% of all cases of CRC, and the fatality rate ranks the fourth and the third for males and females, respectively [2]. Although surgery, chemotherapy and immunity therapy had been developed in the past decade, the prognosis remains unsatisfactory because of the high recurrence rate and metastasis after these treatment. In recent years, a growing study reports that NACRT benefiting in locally advanced patients (T3-4 and/or N+) with rectal cancer (LARC) followed by total mesorectal excision (TME), which has greatly reduced the local recurrence after resection, and increasing the rate of R0 resection [3]. Therefore, NACRT was considered as a standard treatment in LARC patients [4,5]. However, some patients still have poor prognosis for the local recurrence or distal metastatic after surgery. Therefore, there is an urgency to develop reliable non-invasive prognostic predictors for patients with rectal cancer who received NACRT.

Recently, several serological inflammation-based markers included neutrophil–lymphocyte ratio (NLR) [6], platelet–lymphocyte ratio (PLR) [7] and lymphocyte–monocyte ratio (LMR) could help predict prognosis of rectal cancer [8,9]. However, there few studies report the prognostic value of red cell distribution width (RDW) in patients with rectal cancer after neoadjuvant therapy.

RDW is a routine part of a complete blood count test that can reflex the heterogeneity of red blood cell, and correlated with inflammation and nutritional status. Several studies reported that poor nutritional status and cancer-related chronic inflammation could provoke RDW elevation [10–12], and several studies have reported that elevated RDW is related to poor prognosis in several cancers [13–17]; therefore, RDW was likely to be also closely related to the prognosis of rectal cancer. However, the prognostic effect of RDW in patients with rectal cancer that received NACRT remains unclear and needs further research. Thus, the aim of our study was to investigate the prognostic value of RDW to assess and guide clinical treatment to improve the survival of patients with rectal cancer.

## Materials and methods

### Patients

We collected the data of 120 consecutive patients who underwent Mile’s/Dixon (several patients with liver metastases underwent metastatic resection simultaneously) for histologically diagnosed adenocarcinoma of the rectum at Affiliated Cancer Hospital of Zhengzhou University between from June 2013 to May 2015. The inclusion criteria for selected patients were as follows: (1) All patients with rectal cancer have pathological diagnosis using the International Classification of Diseases for Oncology third edition (ICD-O-3) codes (8010–8231 and 8255–8576). (2) All patients underwent NACRT. The mean radiation dose was 50Gy (range 45–55Gy) with daily fraction of 1.8–2.0Gy. Radiation treatments were performed according to the institutional protocols, oral capecitabine was administered at a dose of 1650 mg/m^2^/day daily during the whole period of NACRT. (3) All patients underwent R0 resection after NACRT, and the interval from the completion of NACRT to surgery less than 8 weeks. (4) Six cycles of XELOX chemotherapy were performed after TME. The exclusion criteria were the following: (1) Patients with infection, rheumatoid diseases or other inflammatory conditions. (2) Did not complete NACRT and/or follow up; (3) the interval from the completion of NACRT to surgery more than 8 weeks. (4) Underwent R1 or R2 resection, or died due to non-cancer related causes. The present study was approved by the Institutional Review Board of Henan Cancer Hospital.

### Clinicopathological factors

All the clinical data including age, sex, ECOG score, tumor location, tumor size, pathological differentiation, T stage, N stage, TNM stage (American Joint Committee on Cancer criteria, AJCC criteria 7th edition) [18], metastatic lymph node, vascular invasion, neurological invasion, TRG score, pre-RDW, pre-operative RDW and post- operative RDW were collected by reviewing the medical records before NACRT, surgery and first postoperative chemotherapy, respectively. Surgeries were operated by experienced surgeons of Henan Cancer Hospital. All patients were divided into two groups according to the cut-off value of RDW.

### Follow-up

Patients were followed at 3-month intervals for 2 years, at 6-month intervals for the next 3 years. Recurrence was determined by clinical and radiologic examination or histologic confirmation, these auxiliary examination including colonoscopy, ultrasound, PET-CT, MRI, CT scans for the abdomen, pelvis and chest were performed. The median follow-up was 50 months. Disease-free survival (DFS) was the time from the surgery to the local /distant failure or death. Overall, survival (OS) was calculated from surgery to death induced by cancer-related causes, or end of follow-up.

### Statistical analysis

Continuous variables are expressed as mean ± standard deviation and compared using the Mann–Whitney *U* test. The receiver operating characteristic (ROC) analysis was applied to determine the optimal cut-off values of pre-RDW, pre-operative RDW and post-operative RDW values. The prognostic prediction priority of RDW was compared by areas under the ROC curve (AUC). Survival analysis was conducted using the Kaplan–Meier method and differences between the survival curves were determined by the log-rank test. Multivariate analyses of factors considered, prognostic of overall survival (OS) were performed, using Cox’s proportional hazards model and a stepwise procedure. Clinicopathological factors were included in multivariate analysis. *P*<0.05 was considered significant. Statistical analysis was performed by using SPSS 23.0 software (IBM, U.S.A.). All statistical tests used in the present study were two-sided and *P*<0.05 was considered statistically significant.

## Results

### The baseline characteristics of eligible patients

#### Pre-RDW

**Figure 1:**
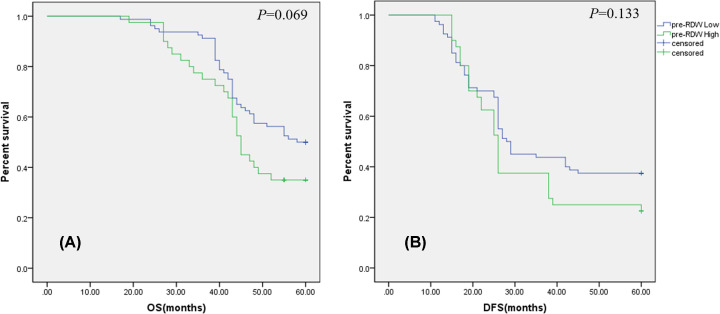
Prognostic value of the RDW prior to NACRT in patients with rectal cancer (**A**) Five-year overall survival (OS) rates have no statistical difference between the pre-RDW^High^ group and pre-RDW^Low^ group (*P*=0.069). (**B**) Disease-free survival (DFS) curves by pre-RDW. Five-year survival rates have no statistical difference between the pre-RDW^High^ group and pre-RDW^Low^ group (*P*=0.133); NACRT, neoadjuvant chemoradiation therapy.

The mean pre-RDW was 13.42 (range 11.70–17.60). Pre-RDW was significantly higher in lower tumor location patients, with T4 stage, with ECOG score ≥ 1, with vascular invasion than in patients with middle-high tumor location (*P*=0.003), with T3 stage (*P*=0.004), with ECOG score = 0 (*P*=0.004), without vascular invasion (*P*=0.005), respectively (Table 1). ROC analysis for 5-year OS (AUC = 0.618, *P*=0.027) and DFS (AUC = 0.639, *P*=0.014) indicated the optimal cutoff value of pre-RDW to be 14.05, 14.00, respectively. Then, patients were divided into the high pre- RDW group (pre-RDW^High^≥14.05; *n*=40) and the low pre-RDW group (pre-RDW^Low^< 14.05; *n*=80) according to 5-year OS, pre-RDW^High^(≥14.00; *n*=39) and pre-RDW^Low^ (<14.00; *n*=71) according to DFS, respectively. However, no relationship was found between pre-RDW and prognosis (OS, *P*=0.069; DFS, *P*=0.133) in our study (Figure 1).

**Table 1:** Comparison of patient characteristics versus pre-RDW

Variables	Pre-RDW	*P* value
Gender		0.139
Female (*n*=44)	13.71 ± 1.87	
Male (*n*=76)	13.25 ± 1.51	
Age (years)		0.793
<60 (*n*=94)	13.41 ± 1.79	
≥60 (*n*=26)	13.48 ± 1.08	
ECOG score		0.004
0 (*n*=85)	13.14 ± 1.61	
≥1 (*n*=35)	14.10 ± 1.62	
ypTumor size (cm)		0.162
≤2 (*n*=58)	13.21 ± 1.58	
>2 (*n*=62)	13.63 ± 1.74	
Histology		0.232
Well-differentiated (*n*=75)	13.28 ± 1.47	
Poor-differentiated (*n*=45)	13.66 ± 1.94	
TRG score		0.218
0-1 (*n*=60)	13.23 ± 1.71	
≥2 (*n*=60)	13.61 ± 1.60	
Tumor location		0.003
Low (*n*=69)	13.79 ± 1.79	
Middle-high (*n*=51)	12.90 ± 1.32	
Depth of invasion		0.004
T3 (*n*=45)	13.08 ± 1.59	
T4 (*n*=75)	13.99 ± 1.65	
ypN stage		0.610
N0 (*n*=62)	13.20 ± 1.94	
N≥1 (*n*=58)	13.73 ± 1.11	
Stage of disease		0.173
II (*n*=61)	13.24 ± 2.01	
III-IV (*n*=59)	13.64 ± 1.10	
Surgical operation		0.617
Mile’s (*n*=46)	13.32 ± 1.65	
Dixon (*n*=74)	13.48 ± 1.68	
Neurological invasion		0.597
Absent (*n*=90)	13.45 ± 1.84	
Present (*n*=30)	13.32 ± 0.96	
Vascular invasion		0.005
Absent (*n*=78)	13.11 ± 1.61	
Present (*n*=42)	14.04 ± 1.61	

TRG score, tumor regression grading score.

#### Pre-operative RDW

**Figure 2:**
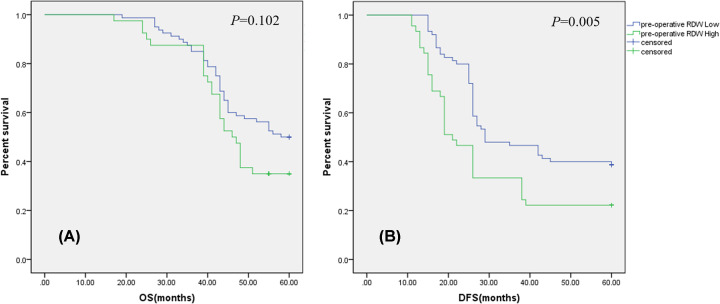
Prognostic value of the RDW prior to surgery in patients with rectal cancer (**A**) Five-year overall survival (OS) rates have no statistical difference between the pre-RDW^High^ group and pre-RDW^Low^ group (*P*=0.102). (**B**) Disease-free survival (DFS) curves by pre-operative RDW. Five-year survival rates were significantly less in the pre-RDW^High^ subgroup than in the pre-RDW^Low^ group (*P*=0.005); RDW, red cell distribution width.

The mean pre-operative RDW was 16.35 (range 12.40–27.60). Pre-operative RDW was significantly higher in patients aged ≥60 years, with T4 stage, with ECOG score ≥ 1, with yp tumor size>2cm, with TRG score ≥2, with stage of III-IV, with neurological invasion than in patients <60 years (*P*=0.022), with T3 stage (*P*=0.005), with ECOG score = 0 (*P*=0.023), with yp tumor size ≤ 2 cm(*P*=0.001), with TRG score 0-1 (*P*=0.002), with stage of II (*P*=0.045), with neurological invasion (*P*=0.01), respectively (Table 2). ROC analysis for 5-year OS (AUC = 0.630, *P*=0.014) and DFS (AUC = 0.616, *P*=0.039) indicated the optimal cutoff value of pre-operative RDW to be 16.45, 16.50, respectively. Then, patients were divided into the high pre-operative RDW group (pre-operative RDW^High^ ≥ 16.45; *n*=40) and the low pre-operative RDW group (pre-operative RDW^Low^ < 16.45; *n*=80) according to 5-year OS, pre-operative RDW^High^ (≥16.50; *n*=45) and pre-operative RDW^Low^ (<16.50; *n*=75) according to DFS, respectively. Pre-operative RDW^High^ group have a worse 5-year DFS (*P*=0.005, Figure 2B) than the pre-operative RDW^Low^ group patients; however, no relationship was found between pre-RDW and 5-year OS (*P*=0.102, Figure 2A) in our study.

**Table 2:** Comparison of patient characteristics versus pre-operative RDW

Variables	Pre-operative RDW	*P* value
Gender		0.211
Female(n = 44)	16.78 ± 2.51	
Male(n = 76)	16.09 ± 3.48	
Age (years)		0.022
<60(n = 94)	15.06 ± 1.38	
≥60(n = 26)	16.68 ± 3.40	
ECOG score		0.023
0(n = 85)	15.33 ± 1.35	
≥1(n = 35)	16.76 ± 3.58	
ypTumor size (cm)		0.001
≤2(n = 58)	15.33 ± 1.87	
>2(n = 62)	17.37 ± 3.81	
Histology		0.090
Well-differentiated(n = 75)	15.97 ± 2.29	
Poor-differentiated(n = 45)	16.98 ± 4.18	
TRG score		0.002
0-1(n = 60)	15.47 ± 1.15	
≥2(n = 60)	17.23 ± 4.15	
Tumor location		0.275
Low(n = 69)	16.08 ± 2.38	
Middle-high(n = 51)	16.72 ± 4.00	
Depth of invasion		0.005
T3(n = 45)	15.31 ± 0.93	
T4(n = 75)	16.97 ± 3.81	
ypN stage		0.132
N0 (n = 62)	15.98 ± 2.56	
N≥1 (n = 58)	16.86 ± 3.81	
Stage of disease		0.045
II (*n*=61)	15.54 ± 2.61	
III-IV (*n*=59)	16.99 ± 3.84	
Surgical operation		0.073
Mile’s (*n*=46)	15.68 ± 1.12	
Dixon (*n*=74)	16.75 ± 3.86	
Neurological invasion		0.01
Absent (*n*=90)	15.05 ± 1.39	
Present (*n*=30)	16.78 ± 3.46	
Vascular invasion		0.556
Absent (*n*=78)	16.23 ± 2.41	
Present (*n*=42)	16.59 ± 4.31	

TRG score, tumor regression grading score.

#### Post-operative RDW

**Figure 3:**
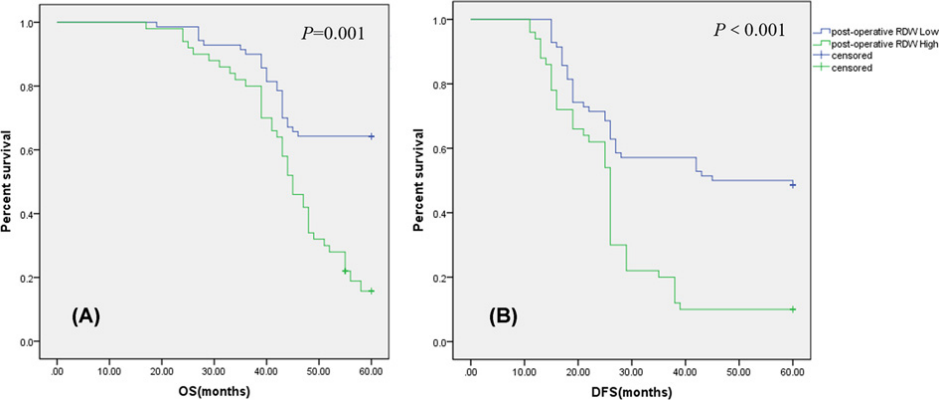
Prognostic value of the RDW after surgery in patients with rectal cancer (**A**) Five-year overall survival (OS) rates were significantly less in the post-RDW^High^ group than in the post-RDW^Low^ group (*P*=0.001). (**B**) Disease-free survival (DFS) curves by post-operative RDW. Five-year survival rates were significantly less in the post-RDW^High^ subgroup than in the post-RDW^Low^ group (*P*<0.001); RDW, red cell distribution width.

The mean post-operative RDW was 15.34 (range 11.70–19.10). Post-operative RDW was significantly higher in female patients, with low tumor location, with N≥1stage, with ECOG score ≥ 1, with yp tumor size > 2 cm, with Mile’s operation, with stage of III-IV, with neurological invasion, with vascular invasion than in male patients (*P*=0.001), with middle-high tumor location (*P*=0.011), with N0 stage (*P*=0.001), with ECOG score = 0 (*P*=0.001), with yp tumor size ≤ 2 cm (*P*=0.021), with Dixon operation (*P*=0.012), with stage of II (*P*=0.001), without neurological invasion (*P*=0.034) and without vascular invasion (*P*=0.002), respectively (Table 3). ROC analysis for 5-year OS (AUC = 0.817, *P*=0.001) and DFS (AUC = 0.862, *P*<0.001) indicated the optimal cutoff value of post-operative RDW to be 15.55 and 15.50, respectively. Then, patients were divided into the high post-operative RDW group (post-operative RDW^High^ ≥ 15.55; *n*=42) and the low post-operative RDW group (post-operative RDW^Low^ < 15.55; *n*=78) according to 5-year OS, pre-operative RDW^High^ (≥15.50; *n*=50) and pre-operative RDW^Low^(<15.50; *n*=70) according to DFS, respectively. Our result suggested that post-operative RDW ^High^ group have a worse 5-year OS (*P*=0.001, Figure 3A) and 5-year DFS (*P*<0.001, Figure 3B) than post-operative RDW ^Low^ group patients.

**Table 3:** Comparison of patient characteristics versus post-operative RDW

Variables	Post-operative RDW	*P* value
Gender		0.001
Female (*n*=44)	16.32 ± 1.55	
Male (*n*=76)	14.75 ± 1.80	
Age (years)		0.210
<60 (*n*=94)	15.23 ± 1.89	
≥60 (*n*=26)	15.76 ± 1.78	
ECOG score		0.001
0 (*n*=85)	14.96 ± 1.74	
≥1 (*n*=35)	16.26 ± 1.88	
ypTumor size (cm)		0.021
≤2 (*n*=58)	14.95 ± 2.04	
>2 (*n*=62)	15.73 ± 1.60	
Histology		0.265
Well-differentiated (*n*=75)	15.48 ± 2.04	
Poor-differentiated (*n*=45)	15.11 ± 1.54	
TRG score		0.285
0-1 (*n*=60)	15.53 ± 1.44	
≥2 (*n*=60)	15.16 ± 2.21	
Tumor location		0.011
Low (*n*=69)	15.71 ± 1.89	
Middle-high (*n*=51)	14.83 ± 1.73	
Depth of invasion		0.281
T3 (*n*=45)	15.56 ± 1.59	
T4 (*n*=75)	15.21 ± 2.02	
ypN stage		0.001
N0 (*n*=62)	14.68 ± 1.58	
N≥1 (*n*=58)	16.27 ± 1.87	
Stage of disease		0.001
II (*n*=61)	14.49 ± 1.59	
III-IV (*n*=59)	16.35 ± 1.68	
Surgical operation		0.012
Mile's (*n*=46)	15.89 ± 1.81	
Dixon (*n*=74)	15.01 ± 1.84	
Neurological invasion		0.034
Absent (*n*=90)	15.13 ± 1.70	
Present (*n*=30)	15.97 ± 2.22	
Vascular invasion		0.002
Absent (*n*=78)	14.95 ± 1.71	
Present (*n*=42)	16.13 ± 1.96	

TRG score, tumor regression grading score.

### The comparison between RDW and the other prognostic indicators

ROC curves were constructed to evaluate survival status, and the AUC values were compared to assess the discriminatory ability of pre- and post-operative NLR, PLR, LMR and RDW (Table 4). The AUC of post-operative RDW was higher than either of the other indicators, indicating that post-operative RDW was the most useful prognostic indicator in rectal patients received NACRT among the indicators included in the current study.

**Table 4:** Receiver operating characteristic analysis of possible prognostic indicators

	AUC	95% CI	*P* value
Pre-prognostic indicators value			
NLR	0.607	0.506–0.709	0.044
PLR	0.515	0.409–0.621	0.782
LMR	0.611	0.502–0.711	0.051
RDW	0.618	0.515–0.720	0.027
Post-prognostic indicators value			
NLR	0.534	0.425–0.642	0.527
PLR	0.499	0.389–0.610	0.987
LMR	0.649	0.551–0.748	0.005
RDW	0.817	0.764–0.909	0.001

Abbreviations: LMR, lymphocyte–monocyte ratio; NLR, neutrophil–lymphocyte ratio; PLR, platelet–lymphocyte ratio; RDW, red cell distribution width.

### Multivariate analysis

Finally, multivariate analysis revealed that post-operative RDW was an independent prognostic indicator for OS, as well as age, TNM stage and post-LMR (Table 5).

**Table 5:** Multivariate analysis of factors considered prognostic of OS in patients with rectal cancer received NACRT using Cox proportional hazard model

	OS					
Factors	Univariate			Multivariate		
	HR	95%CI	*P* value	HR	95%CI	*P* value
Sex (female/male)	0.986	0.965–1.008	0.203	-	-	-
Age (≥60 vs. <60)	0.293	0.179–0.481	0.001	0.903	0.015–0.255	0.001
ypTumor size (cm)	1.460	0.899–2.371	0.126	-	-	-
Histological type well/poor-differentiated	1.620	0.997–2.633	0.052	-	-	-
TRG score (0-1 vs. ≥2)	0.659	0.402–1.081	0.099	-	-	-
ECOG score (0 vs. ≥1)	1.213	0.717–2.052	0.471	-	-	-
Tumor location low versus middle-high	1.836	1.085–3.108	0.054	-	-	-
Mile’s versus Dixon	1.384	0.852–2.249	0.190	-	-	-
pT stage (T3 vs. T4)	0.915	0.557–1.506	0.728	-	-	-
pN stage (N0 vs. N≥1)	4.215	2.546–6.977	<0.001	5.113	3.757–8.745	0.083
pTNM stage (II vs. III-IV)	4.630	2.729–7.855	<0.001	9.566	2.303–39.737	0.002
Neurological invasion (yes vs. no)	1.922	1.136–3.254	0.150	-	-	-
Vascular invasion (yes vs. no)	2.330	1.430–3.795	0.001	9.389	3.561–16.992	0.079
Pre-operative NLR level	1.150	1.005–1.317	0.043	2.501	1.277–4.383	0.067
Post-operative NLR level	0.935	0.818–1.068	0.322	-	-	-
Pre-operative PLR level	0.957	0.903–1.014	0.139	-	-	-
Post-operative PLR level	1.032	0.943–1.131	0.491	-	-	-
Pre-operative LMR level	1.221	1.089–1.370	0.001	2.442	1.478–3.780	0.167
Post-operative LMR level	1.347	1.172–1.549	<0.001	2.388	1.505–3.788	<0.001
Pre-RDW level	1.160	1.025–1.313	0.025	0.64	0.393–1.040	0.072
Pre-operative RDW level	1.062	0.996–1.133	0.065	-	-	-
Post-operative RDW level	1.578	1.373–1.815	<0.001	1.647	1.342–2.201	<0.001

Abbreviations: LMR, lymphocyte–monocyte ratio; NLR, neutrophil–lymphocyte ratio; PLR, platelet–lymphocyte ratio; RDW, red cell distribution width.

## Discussion

RDW has obtained increasing attention in cancer field and elevated RDW was related to poor prognosis in several cancers [19]. The reason of elevated RDW may be the increased inflammation response that induced by cancer cells themselves and cancer microenvironment in cancer patients. An increased cancer-related inflammation response inhibits the generate of erythropoietin, reduces iron release from reticuloendothelial macrophages, and shortens red blood cell survival through relevant inflammatory markers, which results in elevated RDW; however, the potential mechanism has not been demonstrated clearly [20]. In an update of ‘avoiding immune destruction’ and ‘tumor-promoting inflammation’ have been accepted as an emerging hallmark and an enabling characteristic of cancer, respectively [21]. As far as we know that inflammatory bowel diseases and inflammation polys play a crucial role in the development of colorectal cancer [22]. Simultaneously, several studies indicated that several inflammation indicators including NLR, PLR, LMR, interleukin-6 and platelet correlated with the prognosis of colorectal cancer. Above all, we have enough reason to believe that RDW is associated with the outcome of patient with advanced rectal cancer. However, there are have no study systematically reports the effect of perioperative RDW on prognosis in advanced rectal cancer undergoing NACRT.

Surgery is the current treatment for solid tumor include rectal cancer. Several studies have reported that the negative effects of postoperative inflammation on prognosis in patients with colorectal cancer [23]. We have also demonstrated that post-operative LMR was closely related to the rectal cancer prognosis by multivariate regression analysis. These results clearly indicated that post-operative inflammation negatively affected the prognosis of patients with rectal cancer who received NACRT. Therefore, close correlation between post-operative RDW and prognosis observed in our study is likely due to the effect of post-operative inflammation induced by surgery and post-operative cancer-related response. In the present study, we demonstrated that elevation of post-operative RDW was significantly associated with poor prognosis in patients with advanced rectal cancer undergoing NACRT, and investigated the effect of pre-RDW, pre-operative RDW and post-operative RDW systematically on the prognosis after NACRT in patients with rectal cancer. With regard to the usage of post-operative RDW, the prognosis of patients with post-RDW^High^ was significantly worse than that of the patients with post-RDW^Low^ in patients with rectal cancer in our study. We determined that post-operative RDW was useful in predicting the prognosis of patients with rectal cancer than pre-RDW and pre-operative RDW, and suggested that elevated post-operative RDW could predict a poor OS and DFS in patients with rectal cancer after NACRT. Therefore, we proved that the potential ability of post-operative RDW to serve as a prognostic marker for OS and DFS in patients with advanced rectal cancer undergoing NACRT, and potentially representing a noninvasive predictor of prognosis for patients with advanced rectal cancer in the present study.

Pre-RDW, pre-operative and post-operative RDW were closely related to the prognosis by ROC method in the present study. However, multivariate analysis showed that post-operative RDW to be an independent prognostic indicator, but not pre-RDW and pre-operative RDW, and post-operative RDW seemed to be more useful prognostic indicator than pre-RDW, because pre-RDW and pre-operative RDW have no statistical difference in patients with rectal cancer by the Kaplan–Meier method. There is a research reported that elevated pre-RDW can be an independently prognostic factor in patients undergoing resection for non-metastatic rectal cancer [24]; however, the prognostic value of pre-RDW in our report was different from Zhang et al. report in rectal cancer, because received NACRT and several liver metastases patients were enrolled in our study, different inclusion criteria may be the main reason for this difference. Japanese scholar reported that an elevated RDW was an independent prognostic factor for the OS and DFS in patients with LARC undergoing chemoradiotherapy followed by surgery [25], the results have differences from our finding. We consider that the main reasons are as follows: first, several liver metastases patients with rectal cancer were enrolled in our study, the patients included in our study have higher TNM stages than Japanese scholar’ s study, this may lead to difference in results; second, the trend of our pre-RDW results is consistent with the findings of Japanese scholar’s study, if increase the number of enrolled patients may be achieve the consistent results; furthermore, our study analyzes pre-operative RDW and post-operative RDW more systematically and confirms that post-operative RDW has a more accurate predictive value than pre-RDW. There are some indicators related to inflammation including NLR, PLR and LMR have been shown to be closely related to prognosis in patients with rectal cancer [26,27]. In the present study, we demonstrated that post-operative RDW was the most useful prognostic indicator among those indicators in patients with rectal cancer. To the best of our knowledge, our paper is the first to demonstrate the prognostic significance of post-operative RDW in patients with rectal cancer.

There are some limitations in our study. First, post-operative RDW values were measured before first postoperative chemotherapy; however, the best time to measure post-operative RDW remains unclear. Second, our study was a retrospective and single-institution design, which may have led to several forms of bias, and the number of patients in our study was small. Therefore, a prospective multicenter study to validate our findings is warranted.

In conclusion, our study indicates the potential of post-operative RDW as a useful prognostic indicator in patients with rectal cancer received NACRT. Because measuring complete blood count is quick, easy, and non-invasive, post-operative RDW may be a useful prognostic indicator in routine clinical settings.

## Data Availability

The data used to support the findings of the present study are included within the article.

## Abbreviations

CRC: colorectal cancer
LMR: lymphocyte–monocyte ratio
NLR: neutrophil–lymphocyte ratio
PLR: platelet–lymphocyte ratio
RDW: red cell distribution width
